# Cyclin and DNA Distributed Cell Cycle Model for GS-NS0 Cells

**DOI:** 10.1371/journal.pcbi.1004062

**Published:** 2015-02-27

**Authors:** David G. García Münzer, Margaritis Kostoglou, Michael C. Georgiadis, Efstratios N. Pistikopoulos, Athanasios Mantalaris

**Affiliations:** 1 Biological Systems Engineering Laboratory, Centre for Process Systems Engineering, Department of Chemical Engineering, Imperial College London, London, United Kingdom; 2 Department of Chemistry, Aristotle University of Thessaloniki, Thessaloniki, Greece; 3 Department of Chemical Engineering, Aristotle University of Thessaloniki, Thessaloniki, Greece; University of Michigan, UNITED STATES

## Abstract

Mammalian cell cultures are intrinsically heterogeneous at different scales (molecular to bioreactor). The cell cycle is at the centre of capturing heterogeneity since it plays a critical role in the growth, death, and productivity of mammalian cell cultures. Current cell cycle models use biological variables (mass/volume/age) that are non-mechanistic, and difficult to experimentally determine, to describe cell cycle transition and capture culture heterogeneity. To address this problem, cyclins—key molecules that regulate cell cycle transition—have been utilized. Herein, a novel integrated experimental-modelling platform is presented whereby experimental quantification of key cell cycle metrics (cell cycle timings, cell cycle fractions, and cyclin expression determined by flow cytometry) is used to develop a cyclin and DNA distributed model for the industrially relevant cell line, GS-NS0. Cyclins/DNA synthesis rates were linked to stimulatory/inhibitory factors in the culture medium, which ultimately affect cell growth. Cell antibody productivity was characterized using cell cycle-specific production rates. The solution method delivered fast computational time that renders the model’s use suitable for model-based applications. Model structure was studied by global sensitivity analysis (GSA), which identified parameters with a significant effect on the model output, followed by re-estimation of its significant parameters from a control set of batch experiments. A good model fit to the experimental data, both at the cell cycle and viable cell density levels, was observed. The cell population heterogeneity of disturbed (after cell arrest) and undisturbed cell growth was captured proving the versatility of the modelling approach. Cell cycle models able to capture population heterogeneity facilitate in depth understanding of these complex systems and enable systematic formulation of culture strategies to improve growth and productivity. It is envisaged that this modelling approach will pave the model-based development of industrial cell lines and clinical studies.

## Author Summary

The cell cycle is a complex regulatory network that influences not only growth and division, but also other relevant cellular events (e.g. death, productivity, etc.). The development of biologically accurate cell cycle models can help to systematically study mammalian cell cultures. However, the inclusion of segregation in biological systems usually displays a computationally intensive nature. We propose a combined experimental and mathematical framework that allows capturing the heterogeneity in computationally fast and biologically accurate cell cycle models. Using multiparameter flow cytometry a cyclin blueprint is derived to support the model development. Further, the mathematical formulation is reduced to provide a fast solution, allowing its use for sensitivity analysis and model-based parameter estimation. The simulation results are compared to experimental data to test the accuracy and predictive power of the model. This approach can easily be extended to other culture systems, as well as to include further biological detail. The significance of this approach is not limited to industrially relevant cell lines but its application extends to cell cycle relevant systems such as clinical problems (tumours, cancer treatments, etc.).

## Introduction

Monoclonal antibodies (mAb) represent a key growth section of the high-value bio-pharmaceuticals (biologics) market [1]. These biologics are commonly produced by mammalian cell culture systems due to their ability to perform human-compatible post-translation modification (glycosylation) of proteins. Mammalian cells represent complex production systems whereby a large number of interlinked metabolic reactions control productivity and product quality, which are influenced by culture parameters. Mammalian cell cultures are intrinsically heterogeneous at all scales from the molecular to the bioreactor level [2–4]. The key underlying source of heterogeneity is cell cycle segregation [5–7], which is at the centre of cellular growth, death, and productivity, all of which vary during the different cell cycle phases. Specifically, the cell cycle phase can influence the mAb productivity, both of which have been reported to be cell cycle-, cell line-and promoter-dependent [8, 9]. Therefore, a better understanding and knowledge of the cell cycle timing, transitions, and associated production profiles can aid the development (modelling, control, and optimisation) of these industrially-relevant systems [10]. Recently, metabolic flux analysis (MFA) has become a key tool for the study of mammalian cell cultures aiming at improving productivity and product quality. These studies [11–14] provide valuable insight on cell behaviour and assist in understanding cell metabolism. However, they neglect the intrinsic heterogeneity (e.g. cell cycle, genotypic, and phenotypic variations) [15, 16] of cell culture systems. Moreover, MFA applicability to mammalian cells is limited due to their complexity, pseudo steady-state approximation, unbalanced cell growth behaviour, and adaptation to changing environments [17, 18].

Mathematical models can aid the study of the complex mammalian cell culture systems by capturing the heterogeneity of the cell population, both at the biophase and cellular levels. A number of studies have dealt with the development of cell cycle models and the advancement of modelling techniques to capture the segregation of cell cultures. Alas, the development of relevant segregated cell cycle models has proved challenging, particularly due to difficulties in providing quantitative experimental validation. The vast majority of cell cycle models can be classified as cell ensemble models (CEMs) or population balance models (PBMs) [19–22]. CEMs capture heterogeneity by including a large number of single cell models (SCMs) that differ according to key cellular properties. The approach is based on the assumption that a sufficiently large number of individual cells will represent the behaviour of the population. When CEMs describing a small number of cells were coupled to SCMs computation time was small [23, 24]. However, the number of cell-simulated affects both the resolution of the obtained distributions and the computational burden. An alternative to model segregation is based on the population balance equation (PBE), which describes the number distribution of internal cell states as a function of time. This type of formulation allows the incorporation of information on stage-to-stage transition (e.g. cell cycle) and partition of cell material upon cell division. A detailed review of PBEs is available [25]. A series of studies [26–28] on the solution of PBEs in biological systems showed an increased computational solution time when increasing the number of variables (even with an unstructured cell model). Therefore, most of the available PBMs in cell cultures are coupled to unstructured models in order to reduce the computational burden. The computational time is also significantly increased when increasing model complexity, such as number of stages, variables or structure [21, 26, 29, 30].

Two main features describe the existing PBM formulations: age or mass/volume. Early reports used age as the sole distribution variable [31–33]. Later, more complex formulations were reported using age and an additional intracellular property (e.g. product, RNA) [34, 35]. Similarly, relevant mass/volume based PBMs have been reported [7, 29, 36, 37], such as the coupling a PBE with a SCM [30], which demonstrated the computational intensive nature of the PBM-SCM description. Both PBE formulations for the cell cycle transition in mammalian cell cultures, namely age and mass/volume, are non-mechanistic [38–40] and difficult to experimentally quantify.

In mammalian cells, the cell division cycle occurs in sequential distinct phases: G_1_ (first gap), S (DNA synthesis), G_2_ (second gap) and M (mitosis). However, cells can enter a non-proliferating or quiescent state (G_0_) when conditions (internally or externally) are not favourable for DNA synthesis [41]. Three cell cycle checkpoints are reported [42]: at the G_1_-S phase transition, at the end of the S phase, and between the G_2_-M phase transition. The cell cycle is controlled mainly by two proteins, cyclin-dependent-kinases (CDKs) and cyclins [43]. The transition checkpoints are regulated by the varying concentration of the different cyclins, whereas the concentration of CDKs remains in constant excess throughout [44]. Specifically, the transition between the G_1_-S checkpoint has been reported to be regulated by two cyclins, cyclin D and E. Similarly, cyclin B is reported to regulate the cell’s entrance into mitosis [43].

Herein, cell population heterogeneity was captured by formulating a biologically-relevant distributed cell cycle model for the industrial cell line (GS-NS0). The cyclin and DNA distributed cell cycle model utilised cyclin E1 as a marker for the G_1_ phase, DNA content for the S phase, and cyclin B1 for the G_2_/M phase. Each of these markers can be quantitatively experimentally validated. The model was developed using the mathematical modelling framework previously described [45–47]. Specifically, the parameters’ effect on the model output was evaluated by GSA and the identified significant parameters were re-estimated using a set of batch control experiments. The model simulations were compared to independent batch experiment (with varying initial cell cycle distribution and inoculation density) data to test the model’s accuracy and predictive power. The model can be used as a tool for cell cycle-specific (productivity) studies and model-based control and optimisation applications due to its low computational requirements.

## Results

### Model Structure

The proposed model is a multivariable and multistage PBM. The PBM is coupled to an unstructured metabolic model in order to more accurately represent the system. The model is cyclin distributed for the lumped phases—G_1_/G_0_ (Equation 1) and G_2_/M (Equation 3)—and is DNA distributed for the S phase (Equation 2). The model formulation presented does not have boundary conditions since the communication between the stages occurs internally through source and sinks terms [48].

G_1_/G_0_ phase:

$$\begin{array}{l} \frac{\partial N_{G1}\left(cycE,t\right)}{\partial t}+\frac{\partial r_{G1}\left(cycE,s\right)N_{G1}\left(cycE,t\right)}{\partial cycE}=-\Gamma_{G1}\left(cycE,s\right)N_{G1}\left(cycE,t\right)-k_{d}\left(s\right)N_{G1}\left(cycE,t\right)\\ +2\delta \left(cycE\right)\int_{cycB\min}^{cycB\max}\Gamma_{G2}\left(cycB,s\right)N_{G2}\left(cycB,t\right)dcycB \end{array}$$ (1)

S phase:

$$\begin{array}{l} \frac{\partial N_{S}\left(DNA,t\right)}{\partial t}+\frac{\partial r_{S}\left(DNA,s\right)N_{S}\left(DNA,t\right)}{\partial DNA}=\delta (DNA-1)\int_{cycE\min}^{cycE\max}\Gamma_{G1}\left(cycE,s\right)N_{G1}\left(cycE,t\right)dcycE\\ -k_{d}\left(s\right)N_{S}\left(DNA,t\right) \end{array}$$ (2)

G_2_/M phase:

$$\begin{array}{l} \frac{\partial N_{G2}\left(cycB,t\right)}{\partial t}+\frac{\partial r_{G2}\left(cycB,s\right)N_{G2}\left(cycB,t\right)}{\partial cycB}=-\Gamma_{G2}\left(cycB,s\right)N_{G2}\left(cycB,t\right)-k_{d}\left(s\right)N_{G2}\left(cycB,t\right)\\ +\delta \left(cycB-cycB_{S-G2/M}\right)r_{S}\left(DNA=2,s\right)N_{S}\left(DNA=2,t\right) \end{array}$$ (3)

The number distribution (*N_i_=G_1_,S,G_2_*) is the number of cells distributed on a variable for each phase. The PBM is experimentally derived from the cell’s cyclin blueprint [49], which is used to formulate the cyclin intensity functions. The model utilises cyclin E1 (*cycE*, key for the transition from G_0_/G_1_ to S phase), *DNA* (key for the transition between S phase and G_2_/M) and cyclin B1 (*cycB*, key for the transition between G_2_/M phases to G_0_/G_1_). The cyclin growth functions (*r_i = G1,G2_*, Equation 4) denote cyclin (E1 and B1) build-up on each phase (G_0_/G_1_ and G_2_/M) and are responsible for the transition of the cells within each cyclin domain. Similarly, the DNA growth function (*r_i = S_*, Equation 5) denotes the DNA build-up or synthesis in the S phase and is responsible for the transit of the cells within that phase. The cyclin and DNA growth functions (*r_i_*, Equation 4–5) are substrate/metabolite concentration *(s)* dependent. The substrate/metabolite *(s)* stands in general for the main substrates (glucose, glutamate) and metabolite (lactate). Specifically, the growth functions (*r_i_*) are glutamate-dependent (Equation 7). This dependency was introduced since it has been reported that glutamine, or glutamate in the GS system [50], is the main energy-yielding metabolic route [11, 51, 52] with over 90% flowing into the TCA cycle [53]. In addition, the cyclin growth functions (*r_i = G1,G2_*) were also formulated as lactate-dependent since it has been reported [54, 55] that its concentration inhibits cell growth. Lactate dependency was not included in the DNA growth function (*r_i = S_*), since it has been reported to be fairly constant in duration [56]. Therefore, the cells entered the S phase—by means of the Dirac delta function δ*(DNA-1)*—with an initial DNA content and after doubling their DNA, transit occurs immediately to the G_2_/M phase, i.e. Γ*_S_(DNA = 2) equals to infinity*. Cyclin B1 content in the S phase is approximated, as it is not biologically relevant for the S to G_2_ phase transition [43]. Hence, the cells that transit from the S to the G_2_/M phase, enter—by means of the Dirac delta function *δ(cycB-cycB_S-G2/M_)*—with a cyclin B1 content equal to the one observed experimentally between these two phases. It should be noted that in the general case, the S phase must be described by a bivariate probability density function having as internal coordinates the DNA and cyclin B1 content. However, under specific conditions for the rate functions a mathematical dimensionality reduction of the S equation can be achieved as detailed in S1 Text. This dimensionality reduction is essential since it reduces the computational effort of the model by at least 2 orders of magnitude [7, 21, 30], thus permitting its further use for model-based applications (e.g. sensitivity analysis, parameter estimation, control, and optimisation).

The cyclin transition functions are defined as probability of transition between the G_0_/G_1_ and S phases (Γ_*i = G1*_), as well as between G_2_/M and G_0_/G_1_ phases (Γ_*i = G2*_). The cells complete the cycle by giving birth to two daughter cells and enter—by means of the Dirac delta function δ*(cycE)—* the G_1_ phase with a minimum cyclin E1 content. The cyclin transition (Γ_*i = G1,G2*_, Equation 6) is formulated as a step function, which is zero below the respective cyclin threshold for the phases G_1_ (cyclin E1) and G_2_ (cyclin B1), and becomes positive above the threshold, though it is glucose dependent. Similar step transition functions have been recently identified [57] in age-structured models from experimental data. The glucose dependency was introduced [13] since even though glucose is not the growth limiting substrate, it does prolong the stationary phase and delays the decline phase.

The substrate/metabolite (Equation 7) dependencies were formulated observing Monod type kinetics [58, 59]; where *Glu, Glc*, and *Lac* represent the extracellular glutamate, glucose, and lactate concentrations, respectively. The *K_i = Glu,Glc,Lac_* parameters represent the Monod constant, and the exponential factors (*n, m*, and *p*) capture the sensitivity towards the respective substrate/metabolite.

Cyclins growth functions:

$$r_{i=G1,G2}\left(cyc,s\right)=Cycgrow\cdot f_{\lim Glu}\cdot f_{\lim Lac}$$ (4)

DNA growth function:

$$r_{S}\left(DNA,s\right)=DNAgrow\cdot f_{\lim Glu}$$ (5)

Cyclins transition functions:

$$\Gamma_{i=G1,G2}\left(cyc,s\right)=\{\begin{array}{c} 0\\ prob_{i}\cdot f_{\lim Glc} \end{array}\begin{array}{cc} , & below\\ , & above \end{array}\begin{array}{c} threshold\\ threshold \end{array}$$ (6)

Limiting functions:

$$\begin{array}{ccc} \begin{array}{ccc} f_{\lim Glu}=\frac{Glu^{n}}{Glu^{n}+K_{Glu}{}^{n}} & ; & f_{\lim Glc}=\frac{Glc^{m}}{Glc^{m}+K_{Glc}{}^{m}} \end{array} & ; & f_{\lim Lac}=\frac{K_{Lac}{}^{p}}{K_{Lac}{}^{p}+Lac^{p}} \end{array}$$ (7)

The different populations are calculated as follows, see (Equation 8).

Phase populations:

$$phase_{i=G1,S,G2}=\int_{phase\_\min}^{phase\_\max}N_{i}(x_{i})dx_{i}\;\text{where}\;x_{G1}=cycE,\;x_{S}=DNA,\;x_{G2}=cycB\;$$ (8)

Total population:

$$total=phase_{G1}+phase_{S}+phase_{G2}$$ (9)

Cell cycle fractions:

$$f_{i=G1,S,G2}=\frac{phase_{i}}{total}$$ (10)

Viable cells:

$$X_{V}=\frac{total}{V}$$ (11)

Dead cells:

$$\frac{d\left(X_{D}V\right)}{dt}=k_{d}X_{V}V-k_{lys}X_{D}V$$ (12)

The total population is calculated as the sum of all three distributed domains (Equation 9) and the cell cycle distribution is estimated by Equation 10. The viable cell density (*X_V_*, Equation 11) is expressed by dividing over the reactor working volume (*V*). The model operates under the standard assumption of ideal mixing and is presented in batch operation mode. The dead cell population (*X_D_*, Equation 12) is calculated by accounting for the specific death rate (*k_d_*) of the viable cells and the lysis specific rate (*k_lys_*) of the dead cells. The specific death rate (Equation 13) is linked to the extracellular substrate concentrations, particularly glutamate as this is an essential amino acid for the GS system [50]. The glutamate affinity death parameter (*k_dGlu_*) and the exponential factor *(q)*, define the sensitivity of the cell death to glutamate depletion. The growth rate (μ, Equation 14) is calculated based on the cyclin and DNA growth functions. The calculated times of transit for each phase are based on the minimum threshold for transition and the corresponding cyclin/DNA growth functions.

Specific death rate:

$$k_{d}(s)=k_{d\max}{\left(\frac{k_{dGlu}}{k_{dGlu}+Glu}\right)}^{q}$$ (13)

Specific growth rate:

$$\begin{array}{cccc} t_{i=G1,G2}=\frac{cyclin\_threshold}{r_{i=G1,G2}} & ; & t_{S}=\frac{1}{r_{S}} & ; \end{array}\begin{array}{cc} & \mu =\frac{\ln\left(2\right)}{t_{G1}+t_{S}+t_{G2}} \end{array}$$ (14)

The substrate (Equation 15), metabolite (Equation 16), and antibody (Equation 17) reactor mass balances are specified. The biomass yield on glutamate and glucose is represented by (*Y_i = Glu,Glc_*, Equation 15). Lactate was assumed to be produced (*Y_Lac_*, Equation 16) by both glucose and glutamate consumption, as previously reported [11, 12, 51, 53]. In addition, lactate is also consumed (*Q_Lac_*) [14, 55], which in this case was modelled and triggered by the depletion of glutamate. Antibody production (*q_i_*, Equation 17) is specific for each cell cycle phase. A summary of the declared model variables can be found in Table 1.

**Table 1:** Model variables.

**Nomenclature**	**Description**	**Units**
Γ_i_=_G1,G2_	cyclin transition functions	n/a
Γ_E,i_	cyclin E1 transition function on sub-interval i	n/a
Γ_B,k_	cyclin B1 transition function on sub-interval k	n/a
Γ_S_	DNA transition function	n/a
μ	cell growth rate	h^-1^
*cycE*	cyclin E1 distributed content	% cyclin
*cycEmax*	maximum cyclin E1 content used for domain truncation	% cyclin
*cycB*	cyclin B1 distributed content	% cyclin
*cycBmax*	maximum cyclin B1 content used for domain truncation	% cyclin
*cycBmin*	minimum cyclin B1 content used for the domain start	% cyclin
*DNA*	DNA distributed content	DNA relative content
*f_i = G1,S,G2M_*	cell fraction in phase i	n/a
*f_lim_*	Monod kinetics functions	n/a
*Glc*	extracellular glucose concentration	mM
*Glu*	extracellular glutamate concentration	mM
*h_i = E,DNA,B_*	resolution of a sub-interval (bin)	% cyclin or DNA relative content
*Lac*	extracellular lactate concentration	mM
*mAb*	antibody concentration	mg/L
*N_i = G1,S,G2_*	number of cells distributed on a variable for each phase	Cells
*N_E,i_*	number of cells on sub-interval i of domain cyclin E1	Cells
*N_DNA,j_*	number of cells on sub-interval j of domain DNA	Cells
*N_B,k_*	number of cells on sub-interval k of domain cyclin B1	Cells
*n_i = E,DNA,B_*	number of sub-intervals (or bins) within each domain interval	n/a
*Q_i = Glu,Glc_*	substrate consumption rate	mM/cells/h
*r_i_*	growth functions of the distributed variable	h^-1^
*r_E,i_*	cyclin E1 growth function on sub-interval i	% cyc E/h
*r_DNA,j_*	DNA growth function on sub-interval j	DNA/h
*r_B,k_*	cyclin B1 growth function on sub-interval k	% cyc B/h
*total*	total number of viable cells	cells
*phase_i = G1,S,G2_*	number of cells in phase i	cells
*(s)*	substrate/metabolite concentration	mM
*V*	Volume	mL
*X_V_*	viable cell density	cells/mL
*X_D_*	death cell density	cells/mL

Substrates:

$$\begin{array}{ccc} Q_{i=Glu,Glc}=\frac{\mu }{Y_{i=Glu,Glc}} & ; & \frac{ds}{dt}=-Q_{i=Glu,Glc}X_{V}V \end{array}$$ (15)

Metabolites:

$$\frac{dLac}{dt}=Q_{Glc}Y_{Lac}f_{\lim Glu}+Q_{Glu}\frac{Y_{Lac}}{2}X_{V}V-Q_{Lac}\left(1-f_{\lim Glu}\right)$$ (16)

Antibody:

$$\frac{dmAb}{dt}=\left(q_{G1}f_{G1}+q_{S}f_{S}+q_{G2}f_{G2}\right)X_{V}V$$ (17)

The population balance model (Equation 1–3) resembles the McKendrick type epidemic model [60] and in principle it admits large type asymptotic solutions. Nevertheless, these solutions are not possible in case of coupling with the metabolic model. The metabolic model coupling is essential for systems of changing substrate environments, which is the case in industrially-relevant systems (batch & fed-batch cultures). In the general case of studying cell distribution evolution and considering external time dependency (i.e. metabolic model) the complete problem must be numerically solved.

The system of the hyperbolic partial integrodifferential equations (1–3) is discretised in the cell content space using conservative first order finite differences [61, 62]. In this way it is transformed to a system of ordinary differential equations with respect to time, as the model structure is ideally suited to this type of discretization. The code for the solution of the underlying model was originally developed and a detailed assessment was made to ensure compliance with the Courant-Friedrichs-Lewy (CFL) stability condition. Furthermore, it was found that the optimal time step was the one that exactly corresponded to the CFL condition (even better than smaller time steps) since it eliminated numerical diffusion errors. Finally, it was decided to employ the method of lines (ODEs) combined to backwards first order finite differences in gPROMS in order to utilise its advanced non-linear optimisation capabilities for model validation (e.g. parameter estimation) and model-based analysis (global sensitivity analysis). The stability is automatically checked by the ODE integrator by adjusting the time step in gPROMS. The temporal accuracy is guaranteed by the ODE integrator while the spatial accuracy by making a detailed comparison of results for different discretizations. The detailed discretization of equations is presented below:

The interval (*0, cycEmax*) is divided in *n_E_* equal size sub-intervals, the interval (*DNA = 1, DNA = 2*) is divided in *n_DNA_* equal size sub-intervals, and the interval (*cycBmin, cycBmax*) in *n_B_* equal size sub-intervals. Let us define:

$$\begin{array}{cc} \begin{array}{ccc} \begin{array}{cc} h_{E}=\frac{cycE\max}{n_{E}} & ; \end{array} & h_{DNA}=\frac{\left(2-1\right)}{n_{DNA}} & ; \end{array} & h_{B}=\frac{\left(cycB\max-cycB\min\right)}{n_{B}} \end{array}$$ (18)

$$\begin{array}{ccc} \begin{array}{cc} \begin{array}{cc} N_{E,i}\left(t\right)=N_{G1}\left(ih_{E},t\right) & ; \end{array} & N_{DNA,j}\left(t\right)=N_{S}\left(1+jh_{DNA},t\right) \end{array} & ; & N_{B,k}\left(t\right)=N_{G2}\left(cycB\min+kh_{B},t\right) \end{array}$$ (19)

$$\begin{array}{ccc} \begin{array}{cc} \begin{array}{cc} r_{E,i}=r_{G1}\left(ih_{E}\right) & ; \end{array} & r_{DNA,j}=r_{S}\left(1+jh_{DNA}\right) \end{array} & ; & r_{B,k}=r_{G2}\left(cycB\min+kh_{B}\right) \end{array}$$ (20)

$$\begin{array}{l} \begin{array}{ccc} \Gamma_{E,i}=\Gamma_{G1}\left(ih_{E}\right) & ; & \Gamma_{B,k}=\Gamma_{G2}\left(cycB\min+kh_{B}\right) \end{array}\\ \begin{array}{cc} where & \end{array}\begin{array}{ccc} \begin{array}{ccc} i=0,1,2,\ldots ,n_{E} & ; & j=0,1,2,\ldots ,n_{DNA} \end{array} & ; & k=0,1,2,\ldots ,n_{B} \end{array} \end{array}$$ (21)

The discretization of the equation (1) for G_0_/G_1_ phase, equation (2) for S phase, and equation (3) for G_2_/M phase leads to the following system of ordinary differential equations.

G_0_/G_1_ phase:

$$\frac{dN_{E,i}}{dt}=\frac{-r_{E,i}N_{E,i}+r_{E,i-1}N_{E,i-1}}{h_{E}}-\Gamma_{E,i}N_{E,i}-k_{d}N_{E,i}\;i=1,2,3,\ldots ,n_{E}$$ (22)

$$\frac{dN_{E,0}}{dt}=\frac{-r_{E,0}N_{E,0}}{h_{E}}-\Gamma_{E,0}N_{E,0}-k_{d}N_{E,0}+2\frac{h_{B}}{h_{E}}\sum_{k=0}^{n_{B}}w_{k}\Gamma_{B,k}N_{B,k}$$ (23)

S phase:

$$\frac{dN_{DNA,j}}{dt}=\frac{-r_{DNA,j}N_{DNA,j}+r_{DNA,j-1}N_{DNA,j-1}}{h_{DNA}}-k_{d}N_{DNA,j}\;j=1,2,3,\ldots ,n_{DNA}$$ (24)

$$\frac{dN_{DNA,0}}{dt}=\frac{-r_{DNA,0}N_{DNA,0}}{h_{DNA}}-k_{d}N_{DNA,0}+\frac{h_{E}}{h_{DNA}}\sum_{i=0}^{n_{E}}w_{i}\Gamma_{E,i}N_{E,i}$$ (25)

**Figure 1:**
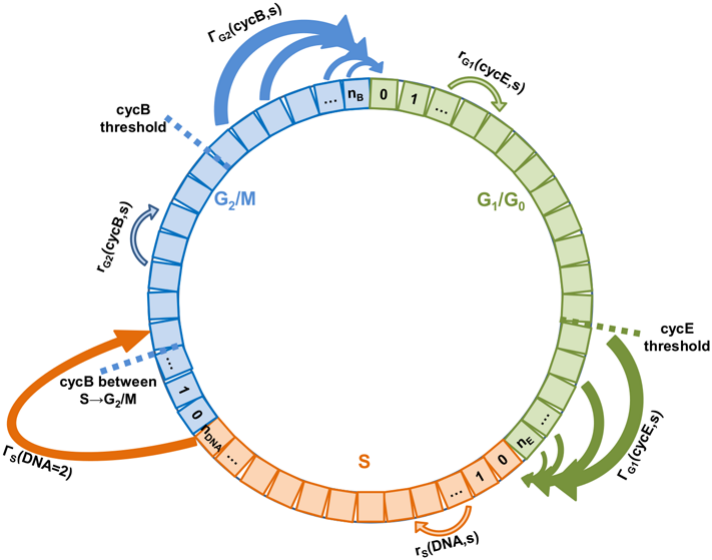
Schematic representation of the discretized cell cycle model.

G_2_/M phase:

$$\begin{array}{l} \frac{dN_{B,k}}{dt}=\frac{-r_{B,k}N_{B,k}+r_{B,k-1}N_{B,k-1}}{h_{B}}-\Gamma_{B,k}N_{B,k}-k_{d}N_{B,k}\\ +\frac{1}{h_{B}}r_{DNA,n_{DNA}}N_{DNA,n_{DNA}}\delta \left(k-\mathrm{int}\left(\frac{cycB_{S-G2/M}}{h_{B}}\right)\right) \end{array}\;\;k=1,2,3,\ldots ,n_{B}$$ (26)

$$\frac{dN_{B,0}}{dt}=\frac{-r_{B,0}N_{B,0}}{h_{B}}-\Gamma_{B,0}N_{B,0}-k_{d}N_{B,0}$$ (27)

where *w_i_* (Equation 23) and *w_k_* (Equation 25) are the weights of the employed numerical integration scheme (trapezoidal). The cyclin B1 content between S and G_2_/M phase (*cycB_S-G2/M_*) is approximated as explained in the S1 Text and the term int(x) denotes the integer part of x. The system of discretized equations (Equation 22–27) is coupled to the metabolic model. A schematic representation of the discretized model is presented (Fig. 1). All simulations were performed using a desktop computer with an Intel Core i7 CPU.

### Cell Proliferation Assay

**Figure 2:**
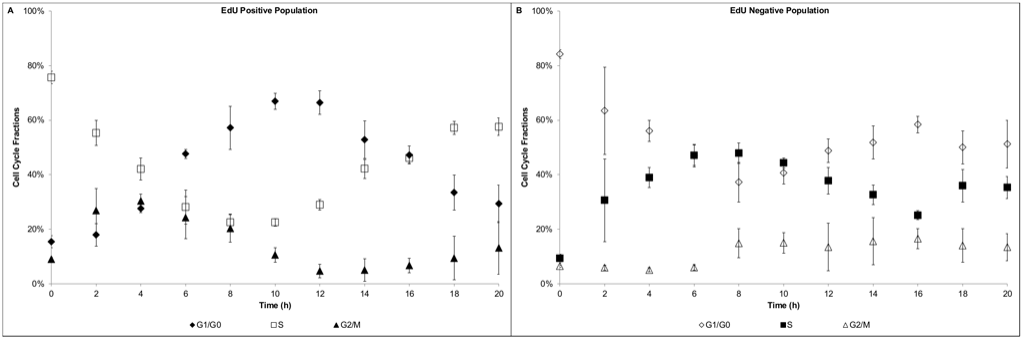
Proliferation assay—Cell cycle distribution. A) EdU positive population; B) EdU negative population.

**Figure 3:**
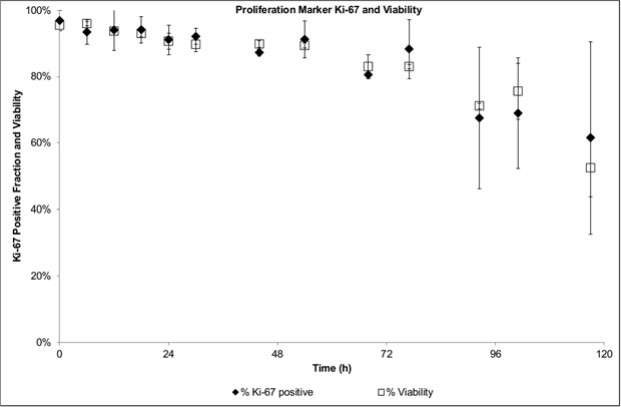
Proliferation assay—Proliferation marker Ki-67 and viability.

Cell proliferation assays provide a direct way to estimate the duration of cell cycle phases. A specific population (which was active during DNA synthesis) of the culture is identified by labelling the DNA base analogue (e.g. EdU) introduced into its DNA. In order to estimate the average transit times based on the DNA content (i.e. G_0_/G_1_, S and G_2_/M phases), EdU positive and negative cells were tracked for each time point of the frequent sampling. The shifts in the distribution of EdU positive cells indicate a fast transit of the cells from S to G_2_/M (Fig. 2A); after 2h of culture a significant increase in the G_2_/M fraction is observed. However, at 2h the fraction of cells at G_1_/G_0_ remains comparable to the starting level, and only after 4h a significant increase is observed. This indicates that the initial EdU positive population traversed the G_2_/M phase in approximately 4h. In order to estimate the G_1_/G_0_ phase time, the EdU positive fraction in the S phase was monitored (Fig. 2A). The S phase was observed to be minimal between 8–10h of culture and soon after an increase was observed (circa 12h). Such increase indicated the completion of the G_1_/G_0_ phase (12h) of the initial EdU positive population (which entered that phase at around 4h); therefore, the estimated timing for this phase is of 8h. The S phase timing can be estimated based on the EdU negative population (Fig. 2B). After 2h, there was a significant change in the S phase, which indicated the entrance of cells into this phase. At around 8h, the number of cells in the S phase peaked with a concurrent increase in the G_2_/M phase fraction. Therefore, the traversing estimate for the S phase is approximately 6h. An average cell cycle time of 18h can be estimated for the GS-NS0 under the specific culture conditions. The estimated value was in agreement with previous reported maximum growth rates of 0.04h^-1^ [63], which corresponds to a doubling time of 17.3h. The cell marker Ki-67 was used to determine the proliferative state of the cell population and discriminate between the G_0_ and G_1_ fractions. A high proliferative state was observed with a greater than 95% positive Ki-67 fraction over 3 days of culture (Fig. 3). After 80h of culture, cell viability started to decline as well as the Ki-67 positive fraction, indicating that some of the cells were entering the non-proliferative state (G_0_). The entrance to the G_0_ phase could not be confirmed to be associated to the commitment to apoptosis (as it was not quantified), however the Ki-67 proliferation marker showed a high correlation with the cell viability.

### Cyclin Profiles

**Figure 4:**
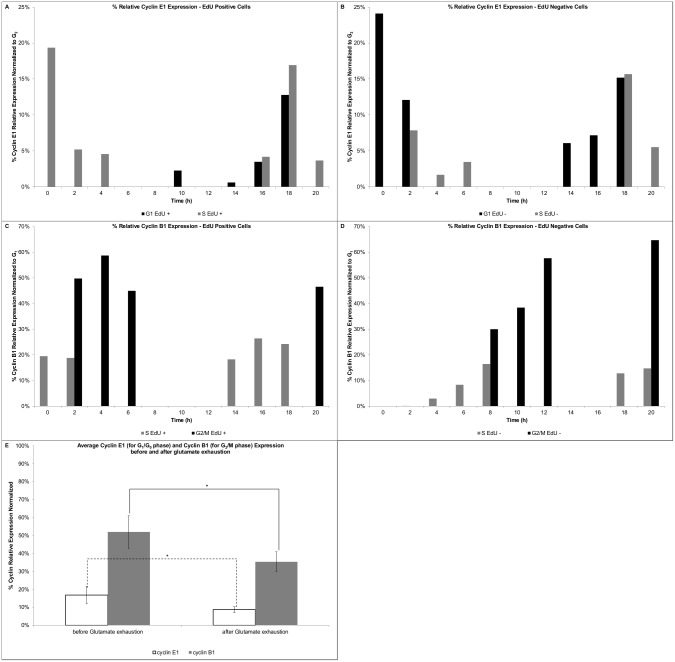
Proliferation assay—Cyclin expression profiles. A) Cyclin E1 expression—EdU positive cells, B) Cyclin E1 expression—EdU negative cells, C) Cyclin B1 expression—EdU positive cells, D) Cyclin B1 expression—EdU negative cells, E) Average cyclin E1 (for G_1_/G_0_ phase) and cyclin B1 (for G_2_/M) expression before and after glutamate exhaustion.

The cyclin profiles for the GS-NS0 cells under similar culture conditions have been recently reported [49], which are in agreement with cyclin expression patterns and thresholds presented herein. By plotting the cyclin content of the EdU positive and negative populations as they traverse the different phases, the cyclin profiles and thresholds could be identified. Cyclin E1 expression profile for the EdU positive population (Fig. 4A) showed the rapid decrease of cyclin E1 expression in the S phase (0–2h), as well as after the G_1_ to S phase transition (18–20h). The cyclin E1 threshold level (between 15–20%) could be identified at the transition (18h). Similarly, the EdU negative population confirmed the cyclin E1 transition threshold (18h, Fig. 4B), as well as the rapid decrease in cyclin expression after the transit (as observed between 0–4h). Cyclin B1 expression profile for the EdU positive population (Fig. 4C), identified the rapid cyclin increase following S to G_2_/M transition at 2h and 18–20h. A comparable profile was observed for the EdU negative population (Fig. 4D). Cyclin B1 build-up in the S phase started at minimum levels to approximately 15% (at 8h). Soon after transition to G_2_/M, cyclin B1 expression builds-up rapidly and peaks between 40–60%, after which cells transit to G_1_. The average cyclin E1 and cyclin B1 expression before (i.e. 0 to approximately 68h of culture) and after (i.e. after 68h of culture) glutamate exhaustion is shown in Fig. 4E. Both cyclins had a significantly lower expression (p<0.05) after the glutamate was exhausted from the media.

### Model Analysis and Parameter Estimation

As outlined in the mathematical modelling development framework [45–47], following the development of the model type and structure, the influence of parameter uncertainty on model output was evaluated. GSA was applied in order to systematically evaluate output variability due to the nonlinear nature of the model. Prior to GSA, the conservation of entities in the system was verified. The birth term was fixed to 1 (to avoid generation), the death rate was fixed to zero (*k_dmax_=0*) and the interaction with the nutrients was neglected (*f_limGlu/Glc/Lac_ = 1*). A uniform distribution was applied for all domains (cyclin E1 between 0–95%, DNA between 1–2, and cyclin B1 between 0–95%). The full range of 24 parameters was varied ±50% [30] from their nominal value and evaluated in over 100 scenarios to test the entities conservation. The nominal values were taken from experimental data (e.g. cell cycle blueprint) and when data were not available, approximated order-of-magnitude values were assigned [30, 63]. The number of bins for the cyclin E1 and B1 domains was increased in order to ensure that over 99% of the entities remained in the system after 120h of simulation. The selected number of bins was 200, both for cyclin E1 and cyclin B1. After fixing the number of bins, GSA was performed on the system. In order to evaluate the relative influence of the uncertainty of the parameters in the outputs, a large number of sampling points is typically required. The parameters were varied ±50% from their nominal value (restoring the birth term, death rate, and limiting functions) and the sampling points were generated using the sobolset function in Matlab. Each scenario was evaluated using the go:MATLAB tool of the gPROMs modelling software. A total of 40,000 scenarios were evaluated and the sensitivity indexes (SIs) were calculated using the GUI-HDMR software [64]. Five outputs were evaluated (*X_v_, f_G1_, f_S_, f_G2_*, and *mAb*) at three simulation time points (36, 72, and 120h). The SIs are the result of the GSA and vary between 0 and 1, with 0 being not significant. To evaluate the results of the GSA a threshold of 0.1 was selected, representing an experimental error of 10% [65]. Therefore, if the SI is above the threshold, the parameter is identified as significant and re-estimated. The non-significant parameters can be fixed at their nominal value.

**Figure 5:**
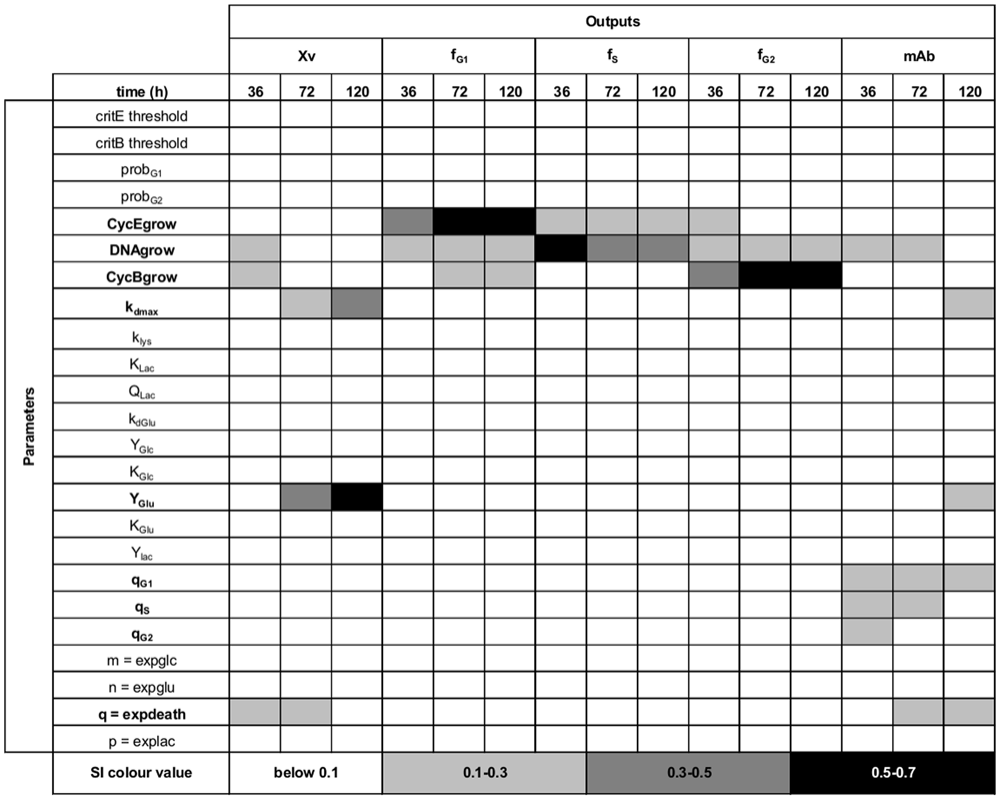
GSA indexes. Outputs: X_V_ = viable cell density, f_G1_ = G_1_/G_0_ cell fraction, f_S_ = S cell fraction, f_G2_ = G_2_/M cell fraction, and mAb = antibody titre.

**Figure 6:**
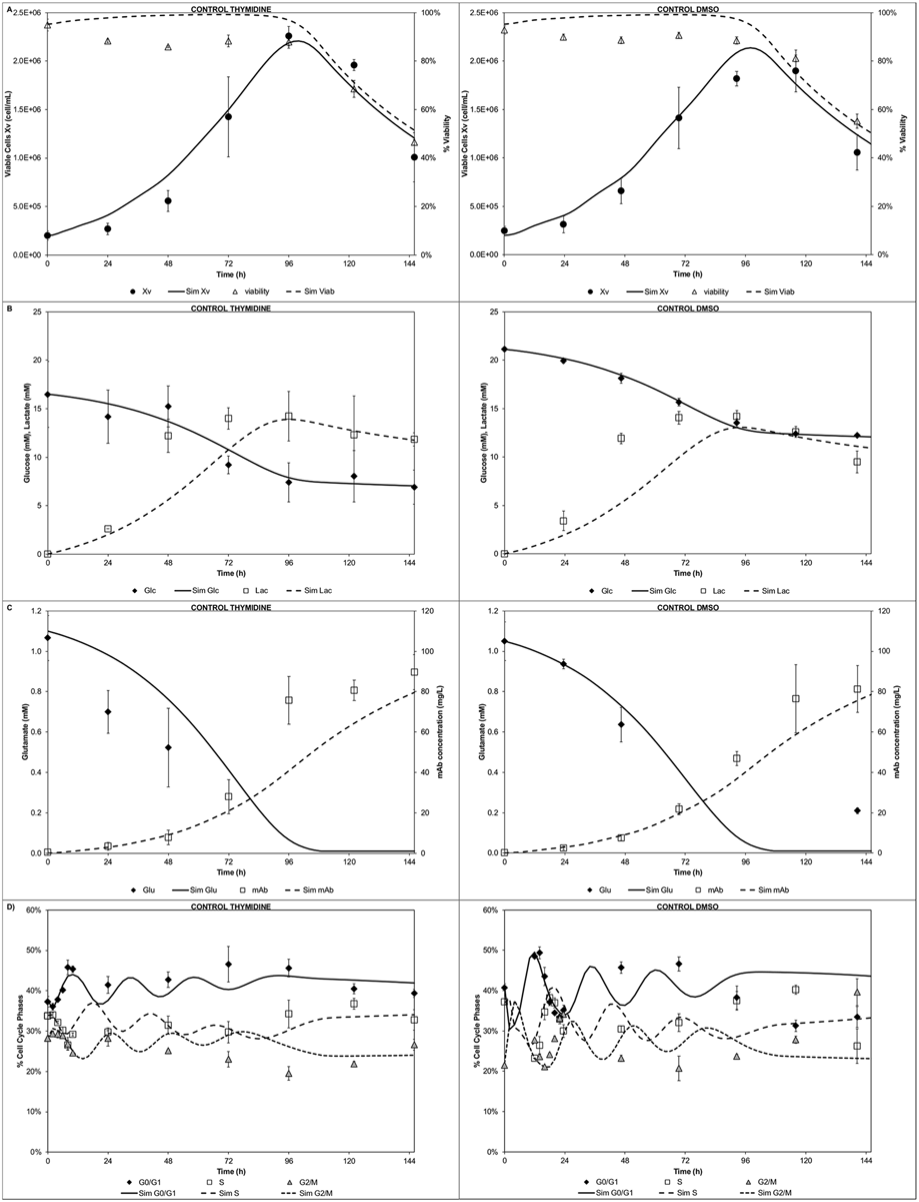
Modelling of control experiments. A) Cell growth and viability, B) Glucose and lactate concentration profiles, C) Glutamate and mAb concentration profiles, D) Cell cycle distribution.

The entities conservation test facilitated the selection of an appropriate number of bins to minimize particle loses during GSA. A trade-off between the number of bins and the solution time is generally observed. Despite selecting a considerable number of bins (i.e. 200) for each cyclin domain and 20 for the DNA domain, the computational time was low (<4s). This enabled completion of the sensitivity analysis (40,000 simulations) in less than 30h using a desktop pc. Both the time for a single run (<4s, two fold faster [7]) and to complete the GSA (<30h) were fast compared to previous reports [7, 21, 26, 30]. Out of the initial 24 parameters, 9 were identified as significant (Fig. 5). The viable cell population output (*X_v_*) was found to be sensitive to the cyclin and DNA growth rates (at early culture time 36h). Also it showed increasing sensitivity (from mid stage 72h towards late stage 120h) to the biomass yield on glutamate (*Y_Glu_*) and maximum death rate (*k_dmax_*). The G_1_/G_0_ cell fraction output (*f_G1_*) showed recurrently (at all stages) sensitivity towards the cyclin E1 growth rate (*CycEgrow*) and DNA growth rate (*DNAgrow*). However, the cyclin E1 growth rate proved to be the most influential parameter for this cell fraction. At mid/late stages also it was found to be sensitive to the cyclin B1 growth rate (*CycBgrow*) stressing the link to the previous cell cycle phase (G_2_/M). The S phase fraction output (*f_S_*) showed a constant and recurrent sensitivity to the cyclin E growth rate for all stages. However, the DNA growth rate was more significant throughout. The G_2_/M cell fraction output (*f_G2_*) showed sensitivity towards the cyclin E1 growth rate at early stage, whereas a constant sensitivity throughout all stages for the DNA growth rate (preceding cell cycle phase) was observed. Also an increasing sensitivity (from early to late stages) towards the cyclin B1 growth rate (*CycBgrow*) was observed. Finally, the antibody titre output (*mAb*) was sensitive to all the cell cycle specific productivities (*q_i = G1,S,G2_*) at early stage (36h). At mid stage it was sensitive to only S and G1 phase specific productivity (*q_S_* and *q_G1_*) whereas at late stage (120h) only to the G_1_ phase specific productivity (*q_G1_*). The identified 9 significant parameters were then re-estimated in order to minimize the uncertainty in the model outputs. Specifically, the control runs for thymidine and dimethyl sulfoxide (DMSO) experiments [49] were used to re-estimate the identified significant set of parameters, while setting the remaining parameters to their nominal value. The model showed good agreement between simulation and experimental results after parameter re-estimation for the cell growth kinetics, peak and decline, as well as the decline in viability (Fig. 6A) for both control runs. The glucose/lactate (Fig. 6B) and glutamate/mAb (Fig. 6C) profiles were also satisfactorily captured. In addition, the model simulated correctly the cell cycle distribution (Fig. 6D) trends, particularly at early stages of the culture. The simulation results were expected to be comparable between the two sets of experiments as the experimental differences are minimal and linked mainly to the initial conditions (cell cycle distribution, centrifugation steps prior to start, glucose concentration [49]).

### Modelling of Arrest Release Experiments

**Figure 7:**
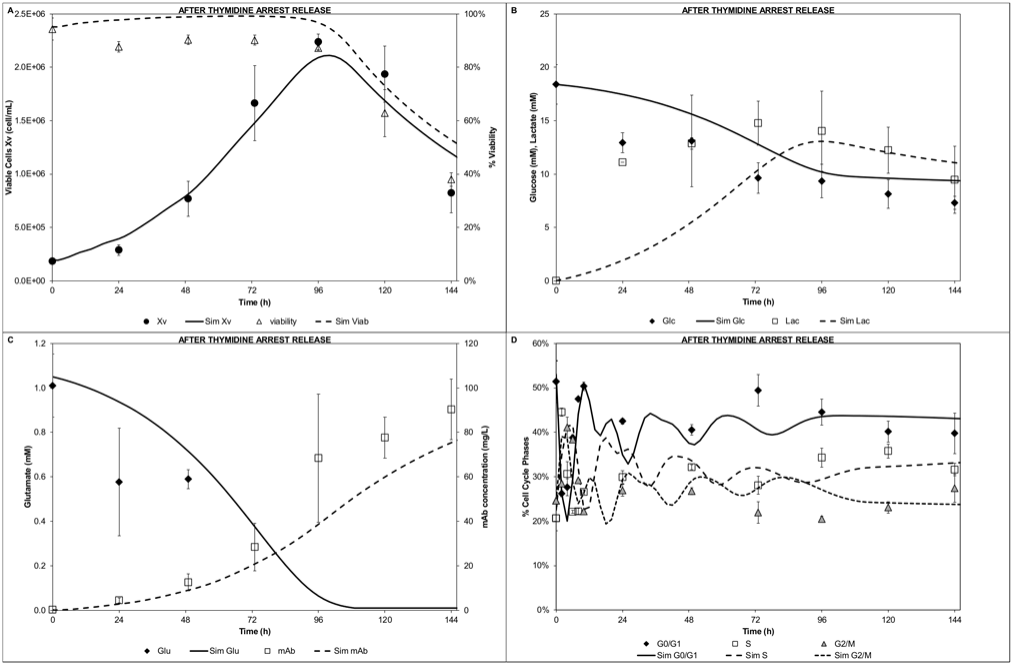
Modelling of after thymidine arrest release experiment. A) Cell growth and viability, B) Glucose and lactate concentration profiles, C) Glutamate and mAb concentration profiles, D) Cell cycle distribution.

The model was then tested against the experiments performed after an arrest release [49]. Although the initial substrates/metabolites conditions were similar to the control runs, the arrest release experiments provided significant different initial cell cycle distribution scenarios. Following thymidine arrest, the cell growth was comparable to the control run, which was reflected by the good model prediction (Fig. 7A). Furthermore, the glucose (Fig. 7B), glutamate, and mAb (Fig. 7C) model simulations showed a good fit. However, the lactate profile (Fig. 7B) showed some discrepancies at early stage (24h) whereas after 48h good agreement was obtained. A plausible explanation to the experimental high lactate levels at 24h has been reported [49]. Nonetheless, the cell cycle distribution (Fig. 7D) showed good agreement with the experimental data. The sharp changes at the early stages of the culture (first 10h), right after the thymidine release, were captured by the model. The good cell cycle fit validated the overall modelling approach and stressed the relevance of the cyclin distribution on the cell cycle transitions.

**Figure 8:**
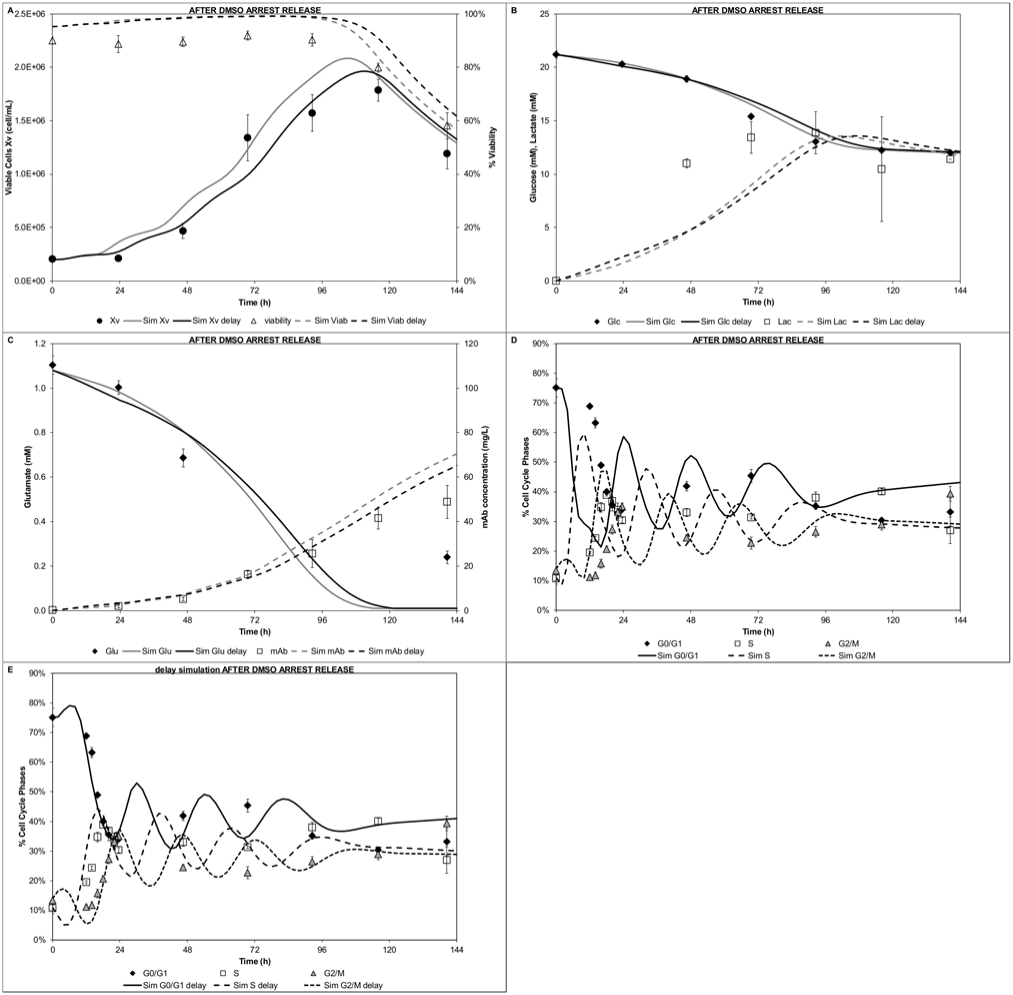
Modelling of after DMSO arrest release experiment. A) Cell growth and viability, B) Glucose and lactate concentration profiles, C) Glutamate and mAb concentration profiles, D) Cell cycle distribution, E) Cell cycle distribution delay simulation. Grey lines: simulation with estimated parameters; Black lines: simulation with DMSO delay parameters (Table 2)

The DMSO arrest release experiment was also modelled. The overall trends for cell growth and viability were in good agreement (Fig. 8A, grey lines), although there was systematic difference between model predictions and experimental data (due to the pronounced experimental lag phase). However, the glucose/lactate Fig. 8B, grey lines) and glutamate (Fig. 8C, grey lines) profiles were satisfactorily predicted. Conversely, the modelled mAb profile (Fig. 8C, grey lines) illustrated a significant deviation from the experimental data (overestimating the final titre). The mAb deviation could be attributed to the higher cell density predicted. Similarly, the cell cycle modelled distributions (Fig. 8D) showed significant differences. Although the trends were correct, the model failed to predict the proper timing to restart proliferation. DMSO has been shown to produce imbalances in the G_1_ cyclins [66]. Therefore, the hypothesis of a slower G_1_ phase completion during the first 24h due to DMSO’s effect was tested in the model. For this purpose, the cyclin E1 growth rate (*cycEgrow*) was decreased. In order to make the simulation comparable to the initial scenario, the biomass yield on glutamate (*Y_Glu_*) and the biomass yield on glucose (*Y_Glc_*) were adjusted by the same factor. The estimated decreased factor for the cyclin E1 growth rate was found to be ~2.3. The three parameters were modified (DMSO delay simulation values, Table 2) for the first 24h of simulation. After 24h, when the effect of DMSO was considered negligible, the modified parameters were set back to their initial values (nominal or estimated values, Table 2). All other model parameters were kept at the reported values. The delay growth simulations showed a marked improvement in the agreement with the experimental data. The cell growth and viability (Fig. 8A) showed an improved fit. Particularly, cell growth and initial lag phase were satisfactorily captured. The glucose/lactate (Fig. 8B) and glutamate (Fig. 8C) profiles showed comparable results to the initial simulation. The modelled mAb titre showed improved prediction when compared to the experimental data (Fig. 8C), although still over predicted the final mAb titre. The slower growth in the G_1_ phase during the first 24h, did seem to improve the cell cycle distribution prediction (Fig. 8E). The model satisfactorily captured the timing for restarting the proliferation and the sharp oscillating cell cycle fractions.

**Table 2:** Model parameters values.

**Parameter**	**Nominal Value**	**Units**	**Estimated Value**	**95% Conf. Interval**	**DMSO delay simulation**
*critE threshold*	15	% cyc E			
*critB threshold*	37	% cyc B			
*prob_G1_*	0.6	n/a			
*prob_G2_*	0.6	n/a			
***CycEgrow***	3.33	% cyc E/h	3.49	1.46E-01	1.52
***DNAgrow***	0.15	DNA/h	0.16	4.76E-03	
***CycBgrow***	8.22	% cyc B/h	8.07	2.99E-01	
***k_dmax_***	3.0E-02	h^-1^	2.31E-02	1.90E-03	
*k_lys_*	5.0E-03	h^-1^			
*K_Lac_*	50	mM			
*Q_Lac_*	2.5E-11	mM/h			
*k_dGlu_*	0.05	mM			
*Y_Glc_*	2.1E+08	cell/mM			9.1E+07
*K_Glc_*	5	mM			
***Y_Glu_***	1.8E+09	cell/mM	1.80E+09	7.20E+07	7.4E+08
*K_Glu_*	0.20	mM			
*Y_lac_*	2.3	n/a			
***q_G1_***	5.0E-10	mg/(cell*h)	9.57E-10	2.10E-09	
***q_S_***	5.0E-10	mg/(cell*h)	1.00E-10	4.30E-09	
***q_G2_***	5.0E-10	mg/(cell*h)	1.00E-11	2.00E-09	
*m = expglc*	2	n/a			
*n = expglu*	1	n/a			
***q = expdeath***	2	n/a	2		
*p = explac*	1	n/a			

### Model Prediction of an Undisturbed Cell Cycle Experiment

**Figure 9:**
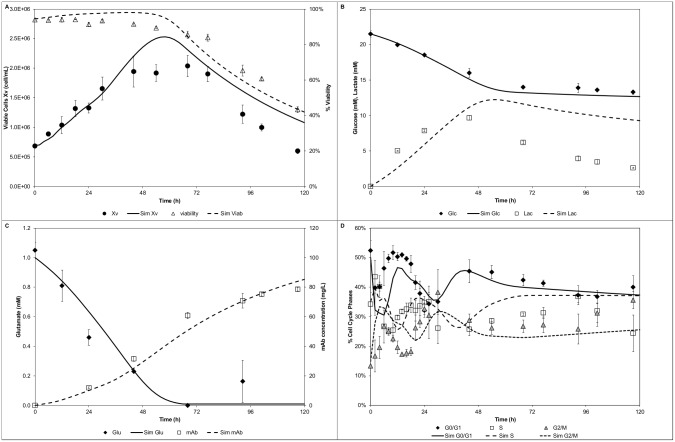
Model prediction of an undisturbed cell cycle experiment. A) Cell growth and viability, B) Glucose and lactate concentration profiles, C) Glutamate and mAb concentration profiles, D) Cell cycle distribution.

An experiment with different starting cell density (2 fold higher) and undisturbed cell cycle distribution was used to test the predictive power of the validated model. The model was able to capture the cell growth kinetics, peak timing, and decline (Fig. 9A). Similarly, viability was closely predicted. Glucose (Fig. 9B) as well as glutamate and mAb (Fig. 9C) profiles were also satisfactorily predicted. At the late stages of the culture (~96h), glutamate seemed to be either produced by the viable cells or released by cell lysis, both of which the model did not account for. The lactate profile (Fig. 9B) showed a good agreement the first 48h of culture whereas at later stages, although the decreasing trend was captured by the model, the decrease in the experimental data was significantly steeper. The observed lactate decrease between 48 and 72h indicated a shift in the cell metabolism. The modelled cell cycle distribution (Fig. 9D) showed a fair prediction. Although the trends were correct a lack of fit was observed during the first 12h, particularly for the G_1_ phase. Despite trying out different initial distribution scenarios, the differences could not be resolved.

## Discussion

The presented model represents a methodical approach to capture the cell culture heterogeneity by including relevant complex cell cycle mechanisms for an industrially relevant cell line. By using the mathematical modelling framework [45, 47, 58, 65, 67] it was possible to evaluate the structure and sensitivity of the model outputs in a systematic manner. Particularly, global sensitivity analysis guided model development and experimentation by identifying key model interactions and parameters, thus reducing experimental costs.

The fact that none of the transition probabilities were identified as significant is not evident. The model uses transition probabilities to determine dispersion around the thresholds. Therefore, as long as there is a positive transition value, the value itself of the parameter is not as significant. It should be noted that if a different model structure for the transition probability was formulated or other outputs were evaluated, it could probably be significant. Similarly, the thresholds for the cyclins were not identified as significant. This does not imply that the transitions are independent of the threshold. The justification is that once a cell reaches the threshold (regardless of its value), the cell becomes a candidate to proceed to the next stage. Therefore, cyclin thresholds and cyclin growth function act as a biologically relevant, mechanistically accurate, and quantifiable substitute to the previously employed variables (e.g. age/mass/volume). Moreover, these markers can be meaningfully coupled with the bioprocessing variables (e.g. substrate availability, chemical arrest agents). In this context, the cyclin blueprint is relevant not for its absolute values of cyclin expression but also in identifying the patterns, timings, and schedule of expression, as this will determine which biomarkers to use in model formulation. Nonetheless, should model validation be extended to cyclin distribution values or considered as an output in the sensitivity analysis, cyclin thresholds might be identified as significant parameters. The relevance of the cyclins and their distribution is evident by their ability to predict cell cycle variations. Finally, the sensitivity of the mAb titre output to the specific cell cycle productivities, although expected, suggests some association. The G_1_ phase specific productivity was the only identified consistently throughout the mAb output as significant. This result suggests a significant correlation of the G_1_/G_0_ phase (compared to the other two phases, i.e. S and G_2_/M) to the total productivity. A similar outcome is observed if the productivity is evaluated in terms of the cumulative cell hours [68] and the analysis is broken down per cell cycle phase [10]. Both a partially growth associated productivity and a better correlation towards the G_1_/G_0_ phase is observed.

Overall, the GSA identified the key parameters that need to be experimentally refined in order to reduce the model’s output uncertainty. This systematic approach is in agreement with previous reports [69] that clearly stress the importance of mathematical models and tools for the analysis and understanding of biological systems.

Model formulation of a PBM coupled to an unstructured metabolic model in conjunction with model reduction demonstrated clear benefits. The computational times were at least two fold faster [7, 28] and enabled the completion of model-based applications, such as GSA, in just over 1 day. This is significantly faster when compared to the 3000h (in a 25 PCs cluster) for the completion of the GSA of a PBM-SCM [30]. The identified significant parameters were re-estimated by tailored made experiments such as the proliferation assay. This experiment allows extracting most of the required data for the model formulation, such as estimated cell cycle times, cyclins’ thresholds, and cyclin/DNA growth rates. Herein, the reported cyclin levels are in agreement with the levels previously reported [49]. The observed cyclin blueprint is cell line and culture condition dependent. These results confirmed the cyclin blueprint without the need to perform any kind of cell synchronisation, and support the cyclin distributed model development. A key aspect included in the model is the link between cyclin expression levels and the culture environment (i.e. substrate and metabolite concentrations). Several reports [70, 71] have addressed the link of the cyclin D family of proteins with the culture environment (growth factors). Although cyclin D is not explicitly formulated in the model, it is expected that cyclin D expression will cascade on the other cyclins (e.g. cyclin E1, B1) and this is explicitly formulated. The experimental data did suggest that the relative expression levels decrease along the batch culture for both cyclins, particularly after the depletion of key energy/anabolic yielding substrates such as glutamate. Nonetheless, due to the employed media formulation (serum-containing), further studies are required. In particular, use of chemically defined media would enable the refinement of the modelled cyclin-extracellular substrate/metabolite concentrations.

The lumped G_1_/G_0_ phases assumption in the model development was supported by a high proliferating cell fraction (Ki-67 positive population). As observed, the cells maintained a high proliferative state as long as key substrates are available. Similarly, the lumped G_2_/M is justified by the short length of the M phase when compared to the other cell cycle phases [72]. However, it should be noted that the cyclin B1 is responsible for the G_2_ to M transition, and herein is approximated to be used for the G_2_/M to G_1_ transition. However, the model can be further refined by segregating the G_2_/M lumped phases (if considered necessary) as M phase markers are also available [73].

The benefits of the presented modelling approach extend to several levels. In first place, the model allows to segregate the population throughout biologically relevant markers. Therefore, the model predictive power is extended to both (un)-disturbed cell cycle conditions, as the cell markers can account for such mechanistic effects. In particular, the impact of the segregation is fully appreciated in the after arrest release experiments. The cyclin segregation provided the right markers to account for the different cell cycle profiles. In particular, after the DMSO arrest release the model did partially capture the experimentally observed delay to restart the proliferation based on the low cyclin E1 expression. More importantly, the model allows us to hypothesize the reason for the delayed start of proliferation and *in silico* postulate a plausible explanation. Herein, by simulating a slower G_1_ transit (capturing indirectly the several mechanistic imbalances caused by the DMSO [66]) for the first 24h *in silico*, was possible to validate and explain the experimentally delayed cell growth, lower peak density and partially account for the lower productivity. Previous models would fail to predict such variations as size/volume cannot account for the delays in the cell cycle transition.

Some model simulations discrepancies were observed in the lactate profiles. Although, the model formulation accounts for the lactate being produced both from glucose and glutamate, it is evident that other sources might be available (e.g. pyruvate readily available in the media [74]). In addition, the included lactate consumption triggered by the glutamate exhaustion showed a fair prediction.

Although, there are no literature reports that directly link lactate consumption and glutamate exhaustion, the selection was based on: 1) in-house experiments of the mid-exponential growing GS-NS0 cells (in the presence of glutamate) showing lactate consumption when cultured on spent medium (without glutamate, S2 Text) and 2) the well-characterised central cellular metabolism that includes glutamate and lactate. The adopted metabolic link herein is supported by studies that report a high conversion of glutamine into lactate [74] and the accumulation of glutamate when lactate is consumed [75]. Similarly, lactate consumption has been correlated to the cessation of rapid growth [55], which occurs in batch mode after the exhaustion of key substrates (such as glutamate) [50]. However, lactate consumption has been shown to be independent of the residual glucose and lactate concentrations in industrial fed-batch cultures [76]. Nonetheless, there is a need to perform additional experiments to confirm the events that trigger such metabolic shifts.

Model prediction of the undisturbed cell cycle experiment showed overall good agreement with the experimental results. However, the need to improve the metabolic description was also noticed. Particularly, a significant deviation was observed in the lactate profile after the glutamate exhaustion, when lactate was noticeably consumed. The observed lactate switch has been reported [14, 55], which as observed has an impact on the proliferation and therefore, on the cell cycle. Specifically, the metabolic switch seemed to be responsible for the lower cell density and provides an opportunity to link key metabolites (carbon source dependent growth rates) to the cell cycle progression. This type of links will help to further develop our understanding of the effect of substrate and metabolites complementing the MFA approach.

Ultimately, the development of cell culture models accounting for the population heterogeneity via mechanistically relevant markers is now possible. The proposed model structure captures the cell population heterogeneity, which can easily accommodate further biological detail (e.g. apoptosis, cell metabolism, energy requirements, and proliferation cessation, among others).

The presented model can be extended to fed-batch processes of industrial relevance. Typically, during fed-batch operation viable biomass, viability, and culture longevity are targeted for production optimisation without considering culture segregation (heterogeneity) [77]. However, the presented model can aid in the selection of optimal proliferation control strategies accounting for culture heterogeneity, which is linked to cell growth limitations, cell cycle distribution, and cell cycle-specific productivity. A key aspect of the presented model is its ability to accommodate for various proliferation control strategies, such as temperature shifts (temperature dependent cyclin/DNA growth rates), variation of medium osmolarity (slowing growth and increasing productivity) [78, 79], and chemical approaches for cell cycle arrest (shown herein). The model could be used to optimise different targets, including the time for arrest induction (either by temperature shifts or chemical addition), maximization of the integral of viable cells (temperature, osmotic effects), or maximizing productivity (temperature shifts, osmotic effects, or chemical addition). Cell cycle segregation relevance in fed-batch processes accounts for cessation of proliferation despite the avoidance of substrate exhaustion. In addition, the model can be coupled to a glycosylation model [80] in order to capture product quality concerns. Several studies have reported changes [81–84], even improvements, on the glycosylation patterns linked to the cell culture environment (e.g. temperature, pH). Although the effects have been attributed mainly to the environmental changes rather than the effects on proliferation [85], the glycosylation coupled model would enable a systematic study of any potential associations between cell cycle and glycosylation. Therefore, the model can accommodate for environmental factors as well as proliferation control strategies, which can include mechanistic effects in the segregated model formulation and cell cycle-specific productivity.

Moreover, the low computational requirements enable the use of the model for model-based applications of industrial relevance such as control and optimisation. Similarly, the used model development framework provides a systematic platform for the development of biological models and quantitatively analysis of the experimental data. The presented approach helps to close the gap between the development of experimental techniques and modelling approaches of cell cultures. Particularly, cell culture systems that need to account for their heterogeneity, such as co-cultures and mixed cultures can benefit from the presented approach. Thus, the methodology can be extended to accommodate more than one cell line/type in describing the population dynamics. The significance of this approach is not limited to industrial scenarios (where the cell cycle productivity or cell growth can be optimised) but also to clinical environments (tumours growth models, design and optimisation of cancer treatments, etc.).

For the first time, to our knowledge, a biologically mechanistic and experimentally quantifiable cell cycle distributed model is formulated. The key role of the cyclins is confirmed at different levels accounting for the proliferative state and as a quantifiable marker. The presented model formulation with its low computational requirements and associated to experimental techniques provide a versatile toolbox that can be further refined to capture the complex heterogeneity and multi-scale nature of mammalian cell culture systems. The developed model satisfactorily predicted different experimental scenarios and can be used as a systematic computer-aided tool to derive optimal operating policies to improve product titre while satisfying tight operating constraints and product specifications. Thus, the overall integrated modelling framework ultimately will pave the development of relevant cell cycle model both at the industrial and clinical environments.

## Materials and Methods

### Cell Culture

Batch cultures over 5 days of GS-NS0 cells (kindly provided by Lonza; passage 5) with expression of a chimeric B72.3 IgG4 antibody were performed as previously described [49].

### Cell Culture Analysis

Cell counts and viability were determined manually using the dye exclusion method (Erythrosin-B; ATCC) and a haemocytometer on a Leica DM-IL inverted phase microscope (Leica). Samples (1.5–2mL) were centrifuged at 800rpm for 5min. The cell-free supernatant was stored at -20°C for metabolic profiling and antibody (IgG4) titre determination. The cell pellets were used for flow cytometry analysis. Metabolic profiles concentrations were measured from the stored supernatant samples with the Nova BioProfile 400 Analyzer (Nova Biomedical). Monoclonal antibody (IgG4) quantification was performed using a high pressure liquid chromatography (HPLC) Agilent 1260 Infinity system (Agilent Technologies). The supernatant was filtered through a 13mm syringe filter with 0.22μm PTFE membrane (VWR, 28145–491) prior to injection. The affinity column, Bio-Monolith Protein A (Agilent Technologies, 5069–3639) was used to quantify IgG4 antibody through the use of a calibration curve. The samples (50μL) were loaded at a flowrate of 1mL/min in a phosphate buffered saline (PBS) mobile phase (pH 7.4) for 2 minutes. Thereafter, a change in gradient was performed using an acetic acid (0.5M, pH 2.6) mobile phase to elute, which was maintained for 5 minutes. Finally the column was again conditioned with a PBS (pH 7.4) mobile phase for 2 minutes. Elution was monitored with a UV detector at 280nm.

### Cell Proliferation Assay

Cells in mid-exponential growth were exposed to 30μM of 5-ethynyl-2 ´-deoxyuridine (EdU, Invitrogen C10419), which is a nucleoside analogue to thymidine and is incorporated into DNA during active DNA synthesis, for a period of 3 hours. After the exposure, the cells were washed twice with PBS and re-suspended in the standard growth media (in the absence of EdU) and cultured in batch mode for 5 days. A parallel control group was established (including all the centrifugation and washing steps without exposure to EdU). Samples were taken every 2h for the first 24h and every 24h afterwards.

### Flow Cytometry

1 × 10^6^ cells were fixed and stored prior to flow cytometric analysis as previously described [49]. Preparation for analysis included removing the fixing agent and washing the cells with a rinsing solution followed by centrifugation, as described [49]. Cell membrane permeabilisation was achieved using 50μL of a saponin-based wash reagent (component E, C10419, Invitrogen) for 15min at room temperature as per the vendor’s instructions. The EdU was detected by adding 0.5mL of the click-IT reaction cocktail (prepared as per vendor instructions). Incubation (room temperature in the dark for 30min) was followed by washing (rinsing solution). The rinsing solution was removed after centrifugation followed by addition of 50μL of a saponin-based wash reagent. Then 20μL of a PE mouse anti-human Ki-67 or the isotype (BD, 556087) were added to stain for the proliferation marker. Incubation (room temperature in the dark for 30min) was followed by washing (rinsing solution). After centrifugation, the rinsing solution was removed and 50μL of a saponin-based wash reagent was added. Antibodies were used for cyclin staining according to supplier instructions as previously reported [49]. Briefly, incubation (room temperature in the dark for 30min) was followed by washing (rinsing solution) and centrifugation. After removing the rinsing solution, 1mL of rinsing solution was added and DNA was stained by adding 1μL of FxCycle violet stain (Invitrogen, F10347). Controls (isotypes, single colours, and isotypes) were also prepared. Flow cytometry was performed on a LSRFortessa (BD) collecting 10,000 events/sample. The following flow cytometry configuration was used: 405nm excitation wavelength and 450/50 filter collection for the violet stain; 488nm excitation and 530/30 filter collection for FITC; 561nm excitation and 582/15 filter collection for PE; 640nm excitation and 670/14 filter collection for Alexa647. FlowJo software (Tree Star Inc) was used for data analysis. Analysis included selection of the cell population from the forward versus side scatter plot, followed by doublet discrimination analysis [49]. Apoptotic cells, debris, and aggregates were gated out from the DNA histogram. Bivariate plots (cyclin vs. DNA) were used to extract the cyclin expression profiles as reported [49]. The proliferating population was gated above the Ki-67 isotype control region (bivariate plots Ki-67 vs. DNA); the G_0_ population was identified in the isotype region. The EdU positive population is gated above the negative EdU population (bivariate plots EdU vs. DNA, cells treated with click-IT reaction cocktail but that were not exposed to EdU).

### Statistical Analysis

All experiments presented were carried out simultaneously in triplicate and the shown values represent the mean ± standard deviation (SD). SigmaStat (Systat Software Inc.) was used for statistical analysis performing analysis of variance (ANOVA) with a level of significance of p<0.05 on data drawn from a normally distributed population. The Kruskal-Wallis ANOVA on ranks with a significance of p<0.05 (Student-Newman-Keuls method) was used when data were drawn from a non-normally distributed population or with unequal variances in order to make multiple comparison.

## Supporting Information

S1 Text Mathematical dimensionality reduction. (DOC)

S2 Text Medium shift experimental data. (DOC)
