# Supplementary material for: Cyclin and DNA Distributed Cell Cycle Model for GS-NS0 Cells
Source: PLoS Comput Biol. 2015 Feb 27;11(2):e1004062. doi: 10.1371/journal.pcbi.1004062 (PMC4344234; doi:10.1371/journal.pcbi.1004062)
Supplement: S1 Text — (DOC) [file pcbi.1004062.s001.doc]

**Appendix S1.** Mathematical dimensionality reduction.

In the general case the S phase probability density function depends both on DNA and cycB. The governing equations take the following form:

| G_1_/G_0_ phase: |  |  |
| --- | --- | --- |
| $\begin{matrix} \frac{\partial N_{G1}\left(cycE,t\right)}{\partial t}+\frac{\partial r_{G1}\left(cycE,s\right)N_{G1}\left(cycE,t\right)}{\partial cycE}=-\Gamma_{G1}\left(cycE,s\right)N_{G1}\left(cycE,t\right)-k_{d}\left(s\right)N_{G1}\left(cycE,t\right) \\ +2\delta\left(cycE\right)\int_{cycB\min}^{cycB\max} \Gamma_{G2}\left(cycB,s\right)N_{G2}\left(cycB,t\right)dcycB \end{matrix}$ | |  |
| S phase: |  |  |
| $\begin{matrix} \frac{\partial N_{S}\left(cycB,DNA,t\right)}{\partial t}+\frac{\partial r_{ScycB}\left(cycB,s\right)N_{S}\left(cycB,DNA,t\right)}{\partial cycB}+\frac{\partial r_{SDNA}\left(DNA,s\right)N_{S}\left(cycB,DNA,t\right)}{\partial DNA}= \\ \delta(cycB)\delta(DNA-1)\int_{cycE\min}^{cycE\max} \Gamma_{G1}\left(cycE,s\right)N_{G1}\left(cycE,t\right)dcycE-k_{d}\left(s\right)N_{S}\left(cycB,DNA,t\right) \end{matrix}$ | |  |
| G_2_/M phase: |  |  |
| $\begin{matrix} \frac{\partial N_{G2}\left(cycB,t\right)}{\partial t}+\frac{\partial r_{G2}\left(cycB,s\right)N_{G2}\left(cycB,t\right)}{\partial cycB}=-\Gamma_{G2}\left(cycB,s\right)N_{G2}\left(cycB,t\right)-k_{d}\left(s\right)N_{G2}\left(cycB,t\right) \\ +r_{SDNA}\left(DNA=2,s\right)N_{S}\left(cycB,DNA=2,t\right) \end{matrix}$ | | |

where all symbols and functions as appeared in the above equations have been described in the main text (reduced model).

A key model approximation can be made, based on: (i) the fact that all cells enter stage S in the same conditions (*DNA=1, cycB=0*) and (ii) the form of functions *r_SDNA_(DNA),* *r_ScycB_(cycB)* which exhibit the same explicit time dependence through the function f_limGlu_. The governing S stage equation is hyperbolic in both internal coordinates so the characteristics along each coordinate can be described by the following ordinary differential equations:

| $\frac{dcycB}{d\tau}=r_{ScycB}\left(cycB,s\right)=CycBgrow\cdot f_{\lim Glu}$ |  |
| --- | --- |
| $\frac{dDNA}{d\tau}=r_{SDNA}\left(DNA,s\right)=DNAgrow\cdot f_{\lim Glu}$ |  |

where τ is the age of the cell in the S stage. Combining the above equations:

| $\frac{dcycB}{dDNA}=\frac{r_{ScycB}\left(cycB,s\right)}{r_{SDNA}\left(DNA,s\right)}=\frac{CycBgrow}{DNAgrow}$ |  |
| --- | --- |

which can be integrated with initial condition *cycB=0* for *DNA=1* to give

| $cycB=FF(DNA)$ |  |
| --- | --- |

The bivariate function *N_S_(cycB,DNA)* can be replaced by the univariate *N_S_(DNA)* using the following relation:

| $N_{S}\left(cycB,DNA,t\right)=N_{S}\left(DNA,t\right)\delta\left(cycB-FF(DNA)\right)$ |  |
| --- | --- |

The error of the above approximation is only restricted to the *cycB*-distribution in S stage and it is related to the initial condition. However, it should be noted that the approximation is exact when all initial S cells abandon S stage i.e. for almost the entire time of practical simulations. Even at the initial time period the error can be very small depending on the initial condition. Therefore the modified problem takes the form of (Eq.1-3) with $cycB_{S-G2/M}=FF(2)$.
