# Supplementary material for: Cyclin and DNA Distributed Cell Cycle Model for GS-NS0 Cells
Source: PLoS Comput Biol. 2015 Feb 27;11(2):e1004062. doi: 10.1371/journal.pcbi.1004062 (PMC4344234; doi:10.1371/journal.pcbi.1004062)
Supplement: S2 Text — (DOC) [file pcbi.1004062.s002.doc]

**Appendix S2.** Medium shift experimental data.

The medium shift experiments consisted on the quick transfer of mid-exponential growth GS-NS0 cells (prior to glutamate exhaustion) to three different medium compositions of fresh and conditioned medium (cell-free, spent medium). The medium compositions consisted on a 100% fresh medium, 50/50% (v/v) fresh/conditioned medium and 0% fresh medium (i.e. 100% conditioned medium). The medium of mid-exponential growing cells was aspirated and the cells were re-suspended in 50mL working volume of one of the levels (carried in triplicates) at a cell density of 5*10^5^ cells/mL. Samples were taken every 6h for 12h, and then at 24h.

| 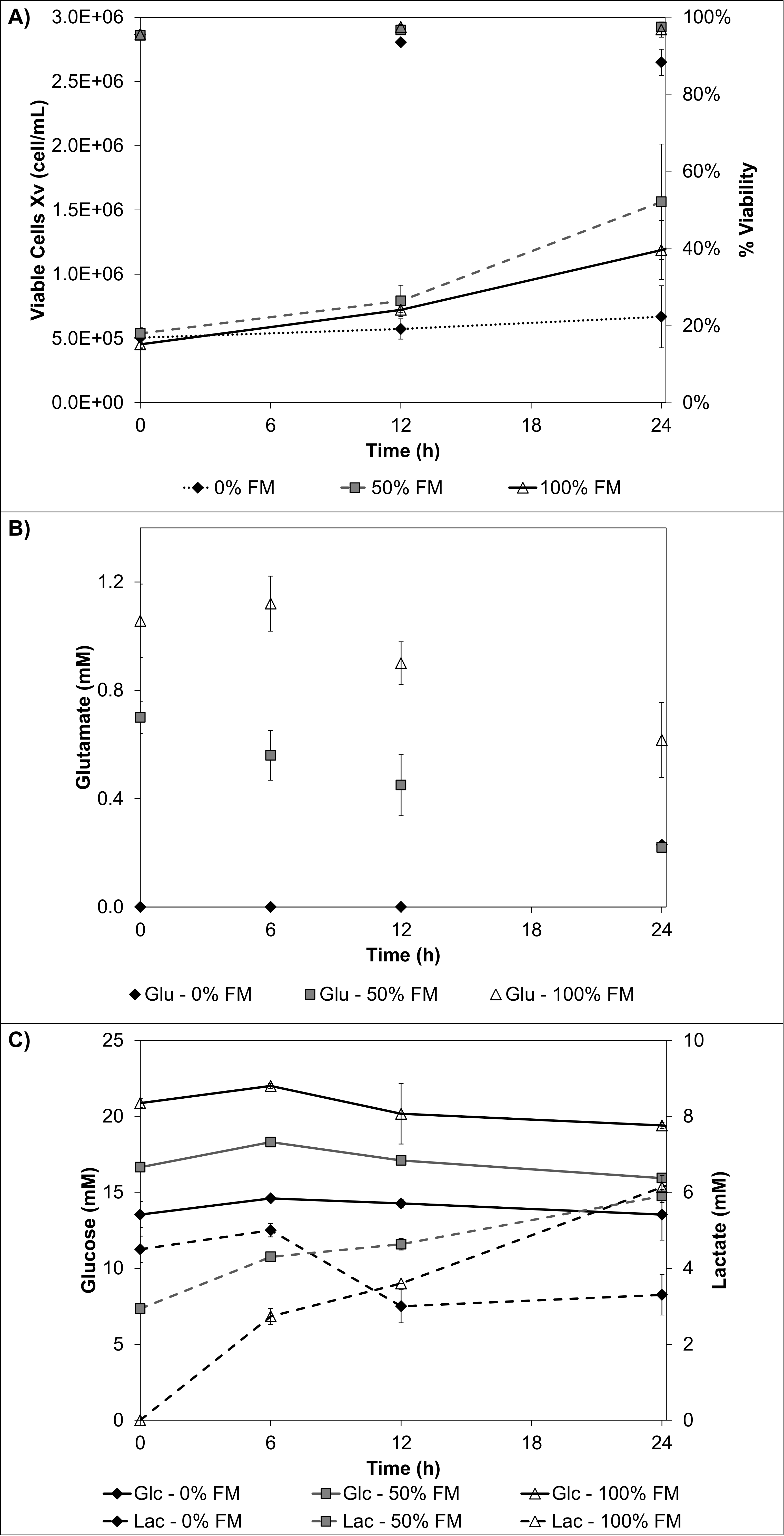 |
| --- |
| **Figure S2.1.** Medium shift experiment profiles. A) Cell growth and viability; B) Glutamate concentration; C) Glucose and lactate concentration. Notation: Fresh medium (FM), viable cells (Xv) with connecting lines, % viability and glutamate without connecting lines, glucose with solid connecting lines, lactate with dashed connecting lines. |

The medium shift experiments showed differences significant differences between the 0% fresh medium and the other two medium compositions on the cell growth and cell viability (FigureS2.1A). Despite all the cultures starting with the same viability, after 24h an approximately 10% decrease in viability was observed in the 0% fresh medium. Glutamate was consumed when present (i.e. 100% and 50% fresh medium) and it was at the limit of detection for the 50% fresh medium after 24h (FigureS2.1B). The glucose profiles of the 100% fresh medium and 50% fresh medium showed a significant glucose concentration decrease between 0h and 24h (FigureS2.1C), as well as a significant increase in the lactate concentration between the same time points. In contrast, glucose consumption in the 0% fresh medium was not evident; whereas a significant lactate decrease was observed between 0h and 24h. Such profiles seemed to indicate a shift in the cell metabolism, going from lactate production (commonly observed) to lactate consumption. This effect has been previously reported in fed-batch cultures [55, 76].
